# Annotations

**Published:** 1902-02-22

**Authors:** 


### Annotations.

### The Medical Faculty and the Academic Council of the University of London.

The Faculty of Medicine after full discussion states that it has come to its knowledge through the Deans of the London medical schools, that a considerable number of young men who desire to study medicine in London and wish to graduate at the University are discouraged from doing so by the character of its matriculation examination. Young men who, having been educated at one or other of the principal secondary schools in this country, come to London with the intention of studying medicine, but, on making inquiries, find that they cannot matriculate at the London University without devoting at least six months to "cramming" for its matriculation examination. In these circumstances many of them elect to be content with a diploma instead of a degree, or betake themselves to some other university where the matriculation is more in accordance with the education which they have received at school. The Medical Faculty evidently feels where the shoe pinches. The supporters of the present system, on the other hand, hold that by maintaining the standard of the matriculation the "duffers" are kept out and the intellectual average of the students is maintained at a high level. If this were the case something might be said for it. The probability is, however, that no such result follows, and that all that the present system does is to very seriously narrow the field from which London University students are drawn, whilst it excludes many intelligent but not specially educated young fellows, and gives an undue advantage to those whose parents and teachers have had the foresight to educate them on those exact but narrow lines which lead to success at this matriculation.

### Sewer Air.

In the early days of sanitation every imaginable evil was attributed to sewer air, and so universal were leaking sewers that the sanitary expert, called in to discover the cause of some puzzling outbreak of disease, generally had an easy task. He must indeed have been a man of small imagination if he could not discover in every cellar some leak by which the dreaded "sewer air" might have found its way to the unfortunate patients upstairs. And so long as "sewer air" maintained its position as the potential parent of all sorts of illness the drain men had a good time. When, however, we arrived at the point of connecting certain diseases with certain bacteria, scientific men not unnaturally asked those who maintained the deadly nature of sewer air to produce their specific bacteria—which they failed to do. Truly it was shown that mice and rabbits when exposed in cages to sewer air, became much more susceptible to various infections than they were when leading an ordinary life, which was not very surprising—but it cannot be denied that for a time sewer air was under a cloud. As a mighty influence in the causation of disease, its glory was departed; it was even held to be less highly charged with microbes than the air of the streets, and infinitely less than that of our day schools. Still, the older among us could not forget the ghastly examples of long ago which we used to regard as proof enough of the evils of sewer air. The fact that specific pathogenic bacilli could not be discovered in it never quite reconciled us to its harmlessness, so that it is with considerable satisfaction that we find the pendulum of science swinging back again once more. Some observations lately made by Dr. Nash upon the bacteriology of the air in the Strand sewer certainly give much support to the idea that such air, especially after rapid motion, is strongly charged with micro-organisms of a distinctly deleterious nature, although possibly not the specific causes of infectious disease.

### Manual Training in the Prison.

We do not often hear in this country of prison labour competing disastrously with free labour, but Mr. Tallack, of the Howard Association, recently found that the free workers in a town he was visiting resented the putting upon the market of work done by the prisoners in the local gaol. As a matter of fact, prison labour does very little harm to free labour, and the competition grievance is a good deal of a bogey, while there is this good reason for prison labour that if criminals are to be made into honest men they must be taught to work. For the average criminal does not know how to work and doesn't want to know. With some knowledge of the use of tools and some education, there is at least the chance of his earning his living honestly when he gets out of prison. But the manual training must be of a general nature, not merely the mechanical doing of one small part of the manufacture of a single article. In our modern methods of industry no one worker makes a whole article, and we know, even among our free workers, how poor are the prospects of advance with the man who can do only one little bit of the process of manufacture compared with him who can turn from one thing to another. A discharged prisoner trained in an equally limited way has certainly not a better chance of earning his living than his non-criminal competitor. If imprisonment is to be reformatory as well as punitive in its character—and nowadays we all desire it to be so—the work taught to the prisoner should be adapted to making it easier for him to stay out of gaol in the future than in the past, rather than to forcing him to contribute to the cost of his maintenance while he is a prisoner. This, in the end, will probably prove the more economic method of the two, for the honest worker is a help to the community, while the criminal, even though he contributes a little towards his own maintenance, is an expense. To make a criminal an honest man is to save money to the taxpayers in the long run. But you can't teach a man a trade in a month or two, even if he is willing, which gaol pupils often are not, at least to begin with. A long sentence is essential to any success in fitting a criminal to become an honest man. Moreover, the prospect of spending three years in gaol, instead of three weeks, or, at the most three months, might set the would-be criminal thinking of the advantages of freedom, even though it had to be purchased by honesty.

